# New York Hospital

**Published:** 1851-11

**Authors:** 


*New York Hospital.* — The medical officers of this noble charity, have done credit to their good sense and humanity, by causing the erection of a spacious tent upon the beautiful green within their enclosure, into which their medical and surgical cases are placed for treatment in the open air, when their condition admits of their removal from the hospital wards, or demands a purer air or better ventilation. Such provision for the sick in hospitals, during the warm season, will be the means of saving human life, as was abundantly shown during the summer of 1847, when 700 cases of ship-fever were thus rescued from perishing in the confined air of the dilapidated buildings then occupied at Bellevue.

This tent at our city hospital, has an elevated plank floor, is open at the sides, and protected from the rays of the sun by a very extended fly, while it is thoroughly lined and otherwise secured against rain. It has been found signally useful, and will doubtless be henceforth adopted among the permanent armamentaria of this hospital. — *N. Y. Medical Gazette.*

